# Adherence of doctors to a clinical guideline for hypertension in Bojanala district, North-West Province, South Africa

**DOI:** 10.4102/phcfm.v7i1.776

**Published:** 2015-04-13

**Authors:** Asafa R. Adedeji, John Tumbo, Indiran Govender

**Affiliations:** 1 Department of Family Medicine and Primary Health Care, Sefako Makgatho Health Sciences University, South Africa

## Abstract

**Background:**

Clinical guidelines are systematically developed statements that assist practitioners and patients to make healthcare decisions for specific clinical circumstances. Non-adherence of doctors to guidelines is thought to contribute significantly to poor delivery of clinical care, resulting in poor clinical outcomes.

**Aim:**

To investigate adherence of doctors in rural district hospitals to clinical guidelines using the South African Hypertension Guideline 2006 as an example.

**Setting:**

Four district hospitals in Bojanala district of North-West Province, South Africa.

**Methods:**

A cross-sectional study determined adherence practices of doctors from records of patients with established hypertension seen at the four district hospitals.

**Results:**

Of the 490 total records documented by 29 doctors, screening for co-morbidity or associated factors was carried out as follows: diabetes mellitus 99.2%, obesity 6.1%, smoking 53.5%, dyslipidaemia 36.9%, abdominal circumference 3.3%; organ damage: eye 0, kidney 82%, heart 43.5%, chronic kidney disease 38.2%, stroke/transient ischaemic attack 15.9%, heart failure 23.5%, advanced retinopathy 0.2%, coronary heart disease 23.7%, peripheral arterial disease 13.9%. Critical tests/measurements were documented in the following proportions: blood pressure 99.8%, weight 85.3%, height 65.7%, body mass index 3.1%, urinalysis 74.5%, lipogram 76.1%, urea/creatinine 80.4%, electrocardiogram 42.9%, blood glucose 100%; risk determination and grading: diagnosis by hypertension severity 19%, low added risk 57.1%, moderate added risk 64.7%, high added risk 89.6%, very high added risk 89.2%. Adherence to therapies was as follows: first-line guideline drugs 69.4%, second line 84.7%, third line 87.8% and fourth-line 89.6%.

**Conclusion:**

Overall adherence of doctors to treatment guidelines for hypertension was found to be low (51.9%). Low adherence rates were related to age (older doctors) and less clinical experience, and differed with regard to various aspects of the guidelines.

## Abstrait

**Adhésion des Docteurs à des directives cliniques pour l’Hypertension dans le district de Bojanala, dans la Province du Nord Ouest d’Afrique du Sud.**

**Contexte:**

Les directives cliniques sont des énoncés systématiquement développés pour aider les médecins et les patients à prendre des décisions sanitaires dans des circonstances cliniques particulières. On les utilise pour normaliser les soins médicaux, promouvoir l’uniformité dans la pratique, améliorer la qualité des soins et réduire les risques liés aux soins cliniques. La non-adhérence des médecins aux directives contribue considérablement à la mauvaise prestation des soins cliniques ce qui entraine de mauvais résultats cliniques. L’étude avait pour but d’examiner l’adhésion dans la pratique des docteurs des hôpitaux de districts ruraux aux directives cliniques, en prenant comme exemple les directives sud-africaines de 2006 pour l’Hypertension.

**Méthodes:**

On a fait une étude dans quatre hôpitaux du district de Bojanala dans la Province du Nord Ouest. On a déterminé l’adhésion aux pratiques des médecins à partir des dossiers des patients souffrant d’hypertension qui ont fréquenté les quatre hôpitaux de district. On a saisi les données sur une fiche de collecte de donnée. On a fait une analyse statistique descriptive sur SAS, Release 9.2.

**Résultats:**

Sur les 490 dossiers documentés par 29 docteurs nous avons trouvé: dépistage de comorbidité ou des facteurs associés: DM 486 patients (99.2%), obésité 30 (6.1%), tabagisme 262 (53.5%), dyslipidémie 181 (36.9%), circonférence abdominale 16 (3.3 %), lésions aux organes: yeux 0, reins 402 (82%), cœur 213(43.5%), CKD 187 (38.2 %), attaque/TIA 78 (15.9%), insuffisance cardiaque 115 (23.5%), rétinopathie avancée 1 (0.2%), maladie cardiovasculaire 116 (23.7%), artériopathie périphérique 68 (13.9%). Les tests critiques et évaluations ont été documentés dans les proportions suivantes: Tension artérielle 489 (99.8%), Poids 418 (85.3%), Taille 322 (65.7%), BMI 15 (3.1%), Analyse d’urine 365 (74.5%), Lipogramme 373 (76.1%), Urée/créatinine 394 (80.4%), ECG 210 (42.9%), GS 490 (100%). La détermination et le degré de risque ont été documentés dans les proportions suivantes: diagnostic par gravité de l’hypertension 93 (19%), risque additionnel faible 280 (57.1%), risque additionnel modéré 317 (64.7%), risque additionnel élevé 439 (89.6%), risque additionnel très élevé 437 (89.2%). Adhésion aux directives thérapeutiques dans 340 (69.4%) médicaments de première ligne 415 (84.7%) de deuxième ligne 430 (87.8%) de troisième ligne, et dans 439 (89.6%) thérapies de quatrième intention.

**Conclusion:**

L’adhésion totale des médecins aux directives de traitement pour l’hypertension est faible (51.9%). Le faible taux d’adhésion est lié à l’âge et à l’expérience clinique du médecin et diffère selon les différents aspects des directives.

## Introduction

Clinical practice guidelines are systematically developed statements that assist practitioners and patients to make healthcare decisions for specific clinical circumstances.^1^ Guidelines can be further defined as a document that streamlines particular processes according to a regular routine.^1^ In the medical context it refers to a document which seeks to guide decisions and criteria regarding diagnosis, management/treatment in specific areas of healthcare.^2^ They are derived from synthesised evidence that have been translated into specific practice-oriented recommendations.^3^ Guidelines identify, summarise and evaluate evidence of the highest quality and the most up-to-date data about prevention, diagnosis, prognosis, therapy including dosages of medications, risk/benefit and cost-effectiveness.^4^

Objectives of clinical guidelines are to standardise medical care and promote uniformity in practice as it relates to care, in order to raise quality of care, reduce several kinds of risk (to the patient, healthcare provider, medical insurers and health plans) and achieve the best balance between cost and medical parameters such as effectiveness, specificity, sensitivity, availability and stakeholders’ preferences. Overall the central role of guidelines is to help clinicians make better decisions.^1^

There has been increasing concern globally regarding poor adherence of clinicians to clinical guidelines. Various reasons have been advanced as barriers to adherence to clinical guidelines amongst medical practitioners.^5,6^ These include the clinicians’ lack of requisite skill and expertise to implement recommended action, lack of the mandatory equipment or staff to implement a guideline recommendation, not being aware of the existence of the guidelines, unfamiliarity or disagreement with the recommendations of the guidelines, lack of confidence in ability to implement the guideline, inability to overcome the inertia of previous practice, the presence of external barriers to following recommendations, a lack of expectancy that adherence to guidelines will lead to the desired process of health care, or simply forgetting to use it.^7^ Poor adherence to clinical guidelines has been reported to contribute to poor health outcomes, compromised quality of care and increased risks, with resultant adverse events.

In the Bojanala district of North-West Province, South Africa, we observed diversity and inconsistency in clinical decisions made by medical practitioners in district hospitals, attributable to erratic adherence to guidelines. In our opinion lack of standardised clinical management of patients may lead to adverse outcomes. Analysis of surveillance data conducted by the Patients Safety Group in North-West Province shows an increasing trend in preventable adverse events resulting from poor adherence of clinicians to guidelines.^8^

A host of clinical management guidelines are readily available in all district hospitals in Bojanala district. Prominent amongst these are management guidelines for hypertension, diabetes mellitus, asthma and epilepsy. As the doctors are not only aware of the existence but also the location of guidelines, it was of interest to the researchers to describe their adherence to guidelines using the 2006 hypertension clinical management guidelines^9^ (still the most recent hypertension guidelines at the time of this study) as a case study.

In the period prior to 2006 there were parallel guidelines produced by the Southern African Hypertension Society and the South African Department of Health, but the 2006 South African Hypertension Guideline is a joint effort by the two bodies.^9^ The guideline outlined the different broad aspects that practitioners need to concentrate on in order to achieve effective blood pressure (BP) control, starting from the initial patient risk screening/profiling, measurements and investigations to be done, risk categorisation and holistic management of hypertensive patients with or without co-morbidities, to their follow up and ongoing care plan.

Our study sought to investigate adherence practices of doctors to the use of clinical/practice guidelines in four district hospitals in Bojanala district, North-West Province. We chose the 2006 Hypertension Guideline as an example as it is freely available at the facilities.

## Research methods and design

A descriptive cross-sectional study using existing patient records was conducted. Records of patients registered as hypertensive in the chronic diseases registers at four rural district hospitals in Bojanala district of North-West Province, South Africa, were evaluated. The four hospitals had 48, 250, 32 and 40 beds, and also conducted outpatient clinics for chronic illnesses, including hypertension. Patient records completed by 29 doctors (general practitioners) at the four hospitals were evaluated.

The formula for calculation of the sample size used was *n* = p (1-*p*) (1.96/a)^2^, where *n* is the sample size, *p* = 0.75 (assumed adherence rate which is 75% [see below] and *a* = 0.04 (accuracy of ± 4%). The 0.75 is halfway between 0.7 (70%) and 0.8 (80%);if the acceptable minimum level of adherence is in the range of 70% – 80%, the precision of the 95% confidence interval, that is the distance from the percentage calculated from the sample to the interval limits, will be ± 4% (which is also the a). This calculation yielded *n* = 450.We oversampled to 484 to allow for missing data, and this was proportionately distributed to the four hospitals as 110, 95, 95 and 184 cases respectively. At each hospital files were consecutively selected until the desired sample size was achieved. Files with missing, illegible or incomplete records were excluded.

The following variables were evaluated for adherence to the hypertension management guideline:

1. Screening for major risk factors: diabetes mellitus, smoking, obesity (body mass index – BMI), dyslipidaemia and abdominal obesity.
2. Grading of risk.
3. Performance of scheduled investigations including fundoscopy, urea and electrolytes, urine dipsticks and electrocardiogram (ECG).
4. Screening for complications, including peripheral arterial disease, heart failure, chronic renal failure, stroke, transient ischaemic attack, advanced retinopathy and coronary heart disease.
5. Routine measurements of the BP (taken at least twice in one consultation, in sitting position), weight, height, BMI, abdominal circumference, urine dipsticks or urinalysis, lipogram, urea and electrolyte levels, ECG and blood glucose level.
6. Documentation of lifestyle modification methods, such as exercise, dietary modification, quitting smoking, alcohol intake.
7. Adherence to prescriptions according to guidelines.
8. Review of treatment and care strategies.
9. Evidence of scheduled review dates.

These data were captured into a data collection sheet. Adherence to each variable was classified as complete (*C*), partial (*P*) or non-adherent (*N*) compared with the reference Hypertension Guideline. Complete adherence referred to an adherence level above 65% and partial adherence to 50 – 64.9%, whereas adherence levels of less than 49.9% were classified as non-adherent. Descriptive statistical analysis was performed using SAS software, version 9.2.

### Ethical considerations

Ethical approval for the study was obtained from the Medunsa Research Ethics Committee (MREC/M/147/2011: PG).

## Results

There were 29 full-time doctors. Doctors in the district hospitals who were considered to have additional qualifications were those with any type of postgraduate medical diploma/fellowship or MMed/college fellowship qualification in Family Medicine. In the four hospitals the mean age of the doctors was 43.9 years (range 29-67 years). There were more males (18) than females (11). The mean length of experience was 15 years (range 4-39 years). More doctors (16) obtained their basic degree locally, whilst 13 had foreign qualifications. Table 1 lists the characteristics of the participating doctors.

**TABLE 1:** Characteristics of the participating doctors.

Variable	Categories						Total (*n*)
Age (years)	**21–30 (%)**	**31–40 (%)**	**41–50 (%)**	**51–60 (%)**	**> 60 (%)**	
		5 (17.2)	11 (37.9)	7 (24.1)	4 (13.8)	2 (6.9)	29
Sex	**Male (%)**		**Female (%)**			
		18 (62.1)		11 (37.9)			29
Qualifications	**Basic medical degree only (%)**		**Medical degree and postgraduate qualification (%)**				
		22 (75.9)		7 (24.1)			29
Where qualification obtained	**Locally (%)**		**Foreign (%)**			
		16 (55.2)		13 (44.8)			29
Years of Experience	**< 5 (%)**		**6–10 (%)**		**> 10 (%)**	
		11 (37.9)		5 (17.3)		13 (44.8)	29

Adherence to screening for major cardiovascular risk factors was high for diabetes mellitus (*n* = 486, 99.2%), moderate for smoking (*n* = 262, 53.5%), low for obesity (*n* = 30, 6.1%), dyslipidemias (*n* = 181, 36.9%) and abdominal obesity (*n* = 30, 6.2%) (Table 2).

**TABLE 2:** Evidence of adherence to the hypertension guidelines.

Variable	Categories assessed	Patient records assessed		
		Adherent (%)	Partially adherent (%)	Non-adherent (%)
Screening for major cardiovascular risk factors	Diabetes mellitus	411 (83.9)	75 (15.3)	4 (0.8)
		Smoking	177 (36.1)	85 (17.4)	228 (46.5)
		Obesity	30 (6.1)	0	460 (93.9)
		Dyslipidaemia	181 (36.9)	0	309 (63.1)
		Abdominal girth	15 (3.1)	15 (3.1)	460 (93.8)
Screening for target organ damage	Eyes	0	0	490 (100)
		Kidneys	402 (82)	0	88 (18)
		Heart	213 (43.5)	0	277 (56.5)
Documentation of measurements done	BP	2 (0.4)	487 (99.4)	1 (0.2)
		Weight	418 (85.3)	0	72 (14.7)
		Height	322 (65.7)	0	168 (34.3)
		BMI	15 (3.1)	0	475 (96.9)
		Abdominal circumference	16 (3.3)	0	474 (96.7)
Documentation of routine investigations	Urinalysis	365 (74.5)	0	125 (25.5)
		Lipogram	373 (76.1)	0	117 (23.9)
		Urea and electrolytes	394 (80.4)	0	96 (19.6)
		ECG	210 (42.9)		280 (57.1)
		Blood glucose	490 (100)	0	0
Documented severity grading	Severity staging of condition according to guideline	93 (19)	0	397 (81)
	Documented care	Lifestyle modification advice was documented	435 (22.2)	345 (17.6)	1180 (60.2)
	Documentation of ongoing care	Referral	385 (78.6)	0	105 (21.4)
		Discussed review date	26 (5.3)	0	464 (94.7)
	Given review date	454 (92.7)	0	36 (7.3)
	Support home visits	2 (0.4)	0	488 (99.6)
Medication	Appropriateness of medication prescribed according to staging	340 (69.4)	0	150 (30.6)
		Review and adjustment of medicine relative to response	256 (52.2)	0	234 (47.8)

Adherence to measurement aspects of the guidelines was high for BP (*n* = 489, 99.8%), whilst adherence to staging of the severity in accordance with the guideline was low (*n* = 93, 19.0%).

Adherence to lifestyle modification/non-drug treatment recommendations was low for physical activity (*n* = 152, 31.2%), dietary modification (*n* = 228, 46.5%), and advice on stopping/reduction of alcohol intake (*n* = 169, 34.5%) and stopping smoking (*n* = 231, 47.2%). Adherence to the ongoing care aspect was high for referral (*n* = 385, 78.6%), whilst adherence to discussion of review date was low (*n* = 26, 5.3%).

When all of the items which were evaluated were considered in totality, overall adherence was found to be 51.9% (Table 3).

**TABLE 3:** Overall adherence to items on the hypertension management guideline.

Hospital	*C* (%)	*P* (%)	*N* (%)	Total items evaluated
A	2323 (47)	249 (5.0)	2373 (48.0)	**4945**
B	1953 (47.3)	208 (5.0)	1967 (47.7)	**4128**
C	3624 (46.3)	388 (5.0)	3813 (48.7)	**7825**
D	1931 (46.4)	248 (6.0)	1984 (47.7)	**4163**
**All hospitals**	**9831 (46.7)**	**1093 (5.2)**	**10137 (48.1)**	**21061**

*P*-value for values from all four hospitals is 0.2623

## Discussion

This study found a generally low adherence of doctors to clinical management guidelines, using the Hypertension Guideline 2006 as an example. Considering all of the different aspects of the guidelines together, the doctors demonstrated complete adherence to 46.7%, partial adherence to 5.2% and non-adherence to 48.1%.

The overall results of our study are comparable to those of another conducted in Pretoria, South Africa, which found that overall adherence to the hypertension practice guideline used by generalists in private practice was 55%, whilst in public service primary care doctors it was 56.4%.^10^

Several determinants of adherence practice, including demographic characteristics of practitioners, training background, experience and the different aspects of the guideline, were investigated in this study. There were more males (62%), foreign qualified doctors (52.2%), those having a basic medical degree (76%), and those with less than 10 years’ experience (56.2%) with average experience of 11.4 years. In addition the doctors were predominantly young, most being in the age range 20–39 years (55.2%), with a mean age of 43.9 years.

The average duration of clinical experience found in this study was comparable to the 12 years’ experience of doctors working in the rural public health sector reported in another study in South Africa.^10^ Evidence from literature points to the fact that doctors’ demographic characteristics affect their attitude to the uptake and adoption of guidelines.^11^ Some of these characteristics are number of years since graduation, age of the doctor, participation in postgraduate education, where the medical degree was obtained, and additional qualifications.^10,12^ Another study on adherence to hypertension clinical guidelines in Saudi Arabia found that 98.1% of rural district primary care physicians were foreign-trained, which was postulated to have influenced their hypertensive guideline adherence.^12^

With regard to adherence specific to various aspects of the hypertension guideline, our study demonstrated no significant interhospital variations. Excluding screening for diabetes mellitus as a major risk factor and screening for target organ damage in the kidney, which showed high (83.9% and 82% respectively) adherence at district level, all of the other variables revealed poor adherence to guideline recommendations. The study from Saudi Arabia reported high to very high (from 88.8% to 99.4%) adherence to all aspects of screening for major risk factors and target organ damage, which agrees only in small part with the present study. Another South African survey of hypertensive patients found much lower adherence than our study, in that doctors reported silent renal disease in 6% of female patients and left ventricular hypertrophy in 35% of the patients.^13^

Adherence to the guideline recommendations on generic parameter measurements and investigations also illustrated poor utilisation of recommendations on BP measurement. Only one BP reading was taken in sitting position in 99.4% of the patients, and BP was taken in both sitting and standing positions in only 0.4% of patients, consistent with partial adherence and complete non-adherence respectively in the present study. This is comparable with the results of the Saudi Arabia study, which established that doctors only measured BP in both sitting and standing positions in 5.6% of patients.^12^ This similarity may be explained by comparable doctors’ profiles in the Saudi and the current study. Another study in Europe also found low adherence (not quantified) to BP measurement guidelines.^14^ Inconsistencies in adherence to BP measurement requirements raise questions about the reliability of hypertension diagnosis.^15^ In one study all patients (100%) reviewed had defective BP measurements taken.^15^ This finding agrees with low adherence figures of the current study on BP measurement, but differs in the type of error committed. Our study confirmed findings from other studies that found that doctors do not measure BP correctly.

The BP and other measurements, such as weight, height, urine dipsticks and blood glucose, are usually done by the nursing staff before the doctors’ consultation with the patients, and they were all done at a high level of compliance. Adherence to performance of other tests/measurements like BMI/abdominal circumference (3.1/3.3% respectively) and ECG (42.9%) were low. The performance of ECGs in our study concurs with the finding of Ardery et al.^16^ (41.5% adherent). The findings of the current study contrasts with the results of the study by Al-Gelban et al.,^12^ which reported that doctors calculated BMI in up to 90.1% and performed ECG in 87.9% of patients that they managed, whereas adherence to lipogram (89.8%), urea and electrolytes (88.2%) and urine analysis (95.7%) measurement in the same study were high to very high, consistent with those in our study. The variation in results between Al-Gelban study and the current study might be due to differences in data collection methods. The Al-Gelban study employed a standardised questionnaire for data collection, which may have made it possible to gather more information about doctors’ adherence; the record review employed by the current study was limited by the quality of documentation.^12^ Conversely, a South African study reported that urinalysis was performed in 49.7% of patients, ECG was not done at all (100% non-adherence rate), whilst BMI and waist circumference were calculated in almost all the patients in the study.^15^

Adherence to the guideline recommendations on diagnosis according to hypertension staging or degree of severity/normality and grading of risk was very low in all the four hospitals (19% for the whole district). This is comparable to the Saudi Arabia study, which reported that 25% of doctors surveyed could not grade the risks of hypertension correctly in the general population.^12^ This similarity may again be explained by comparable doctors’ profiles in the Saudi study and the current study. Adherence to grading of risk aspect of the guidelines was either average or high in all four hospitals (district totals of 57.1%, 64.7%, 89.6% and 89.2% for low added risk, moderate added risk, high added risk and very high added risk respectively). This is comparable to the findings of an European study where 68.5% of participating physicians used a global risk assessment tool whilst another 69.4% reported that they used charts to grade risk.^17^ The tool and the charts were designed using information contained in the 7th Joint National Committee hypertension guidelines from which the 2006 South African Hypertension Guideline was adapted. This may explain the similarity in these findings.

Assessment of documented practice on guideline recommendations regarding the management aspect also demonstrated poor adherence to all four components of lifestyle modification. There were low adherence levels for dynamic exercises (3.1%), dietary modification (21%), advice on stopping smoking (37.8%) and stopping or reducing alcohol intake (26.9%). These findings concur with results from other studies^14,18^ which reported low adherence to recommendations on lifestyle modification and non-pharmacological management. In contrast the Saudi study reported adherence to recommendations on dietary modification at 98.8%, but this study did not address other components of lifestyle modification.^12^

This study revealed generally high adherence to drug treatment recommendations. Adherence to the prescription of first-line treatment (diuretics) when indicated was found to be 69.4%, which is higher than reported in another South African study which found 58.8% adherence to prescription of diuretics by public service doctors when indicated.^10^ Other studies reported reduced use of diuretics,^12,18^ particularly in countries with a trend of increased use of second-line drugs such as angiotensin-converting enzyme inhibitors (ACEIs) and calcium-channel blockers (CCBs).^19^

Adherence to prescription of second-line treatment (ACEI) when indicated was also high, at 84.7%. This is also in keeping with the findings of the Pretoria study, which reported 73.5% adherence amongst public service doctors in patients with compelling indications.^10^ Contrasting adherence data were reported by the Saudi Arabia study (17.7%).^12^ Adherence to prescription of third-line treatment (CCB) when indicated was also high, at 87.8%. This contrasted with both Ernst's^10^ and the Saudi study,^12^ which reported 26.1% and 13.0% adherence respectively to CCB prescription when indicated. Adherence to prescription of a fourth-line treatment (beta blocker) when indicated/necessary was also found to be high, at 89.6% for the district. This correlated with results of the study by Ernst,^10^ which reported a high adherence (73.5%) by public service doctors to use of beta blockers when indicated. In contrast, the study by Al-Gelban *et al.* reported a low adherence of 47.2%.^12^

The present study demonstrated average adherence (52.2%) to drug/treatment review/adjustment which is comparable with results of the study by Ardery,^16^ that reported medication dose adjustment in 55.9% of applicable cases. The present study differs from the study by Berlowitz et al.,^20^ which reported only 25.6% adherence to treatment review when indicated.

Adherence in terms of appropriate referrals of patients was generally high, with values generally ≥ 75%. The adherence figures of medical records, where review dates were discussed and given, were incongruous with each other – review dates were almost always given (adherence figures > 85% in all four hospitals), whilst adherence to discussion of review date was low (less than 10% – extremely low for all four hospitals). This may be attributed to poor documentation on the part of the doctors, although Dickerson et al.^14^ also reported low adherence to follow-up appointment dates. Almost no doctors followed guideline recommendations on ongoing care for follow-up of hypertensive patients (district non-adherence figures stood at 99.6%) in the hospitals.

## Limitations of the study

The confined setting of the study in a single district limits the generalisability of the findings to other settings dissimilar to this one.

## Conclusion

This study found that doctors in rural district hospitals of North-West Province generally have poor adherence to clinical guidelines. Adherence rates to guidelines are related to the age and clinical experience of the practitioner, and differ with regard to the various aspects of the recommendations. There is a need to enhance some of the practical skills necessary for appropriate screening in order to improve adherence to guidelines and improve clinical outcomes.
